# The Pharmacological Potential of *Marantodes pumilum*: A Comprehensive Review of Its Medicinal Properties

**DOI:** 10.3390/ijms26136155

**Published:** 2025-06-26

**Authors:** Siti Hajar Adam, Nor Syaza Syahirah Amat Junaidi, Shariff Halim, Nurul Farisha Ramli, Mohd Helmy Mokhtar

**Affiliations:** 1 Pre-Clinical Department, Faculty of Medicine and Defense Health, Universiti Pertahanan Nasional Malaysia, Kuala Lumpur 57000, Malaysia; siti.hajar@upnm.edu.my (S.H.A.); norsyaza@upnm.edu.my (N.S.S.A.J.); nrulfrisha@gmail.com (N.F.R.); 2 Faculty of Health Sciences, Universiti Teknologi MARA Cawangan Pulau Pinang, Kampus Bertam, Kepala Batas 13200, Malaysia; halimshariff@uitm.edu.my; 3 Department of Physiology, Faculty of Medicine, Universiti Kebangsaan Malaysia, Kuala Lumpur 56000, Malaysia

**Keywords:** *Marantodes pumilum*, pharmacological properties, phytoestrogen, cardiovascular health, anti-inflammatory

## Abstract

*Marantodes pumilum* (MP) is one of the traditional plants to which various medicinal properties are attributed. Studies on the medicinal properties of MP and its characteristics are becoming more extensive and are attracting more and more attention. In this review, the findings on the pharmacological properties of MP have been summarised and analysed. The results show that in addition to its phytoestrogenic effects on the female reproductive system, MP also has bone-remodelling properties, anti-obesity, anti-cancer, anti-gout, antimicrobial, anti-inflammatory and wound-healing effects, as well as effects on the cardiovascular system. These findings show that MP has great potential for the prevention and complementary treatment of various diseases. However, further research is needed to explore its full clinical potential.

## 1. Introduction

For thousands of years, medicinal plants have formed the foundation of traditional healing practises in various cultures, with their bioactive compounds playing a crucial role in the treatment of a wide range of diseases. This rich ethnopharmacological heritage reflects one of the earliest examples of how people used empirical knowledge for therapeutic purposes, before the advent of modern science [1]. Among these botanicals, *Marantodes pumilum* (Blume) Kuntze (MP), formerly known as *Labisia pumila* (Blume) Fern.-Vill, has emerged as a plant of therapeutic interest in the traditional medicine of Southeast Asia. *MP* belongs to the Primulaceae family and is known by various vernacular names including Kacip Fatimah, Selusuh Fatimah, and Rumput Siti Fatimah. The indigenous communities of the Malay Archipelago have utilised this herb for centuries, earning it the title “Queen of Herbs” in regional healing traditions [2]. While this title primarily reflects its traditional role in women’s reproductive health, indigenous knowledge encompasses a much broader therapeutic spectrum, including treatments for gastrointestinal, inflammatory, and infectious diseases [3].

The extensive traditional use of MP has provided a foundation for scientific investigation, as researchers have begun to explore its diverse biological effects beyond reproductive health. MP exhibits a wide range of pharmacological properties, including antioxidant, anti-inflammatory [4], osteoprotective, anti-obesity, cardioprotective [5], antibacterial [6], phytoestrogenic, and anticarcinogenic effects [7], suggesting its potential for various therapeutic applications. Among these properties, the phytoestrogenic effects of MP have attracted scientific and commercial attention, leading to the development of standardised extracts marketed as natural remedies for menopausal symptoms and for the general well-being of women [8]. These effects are attributed to the plant’s phytoestrogens, which, due to their structural similarity to endogenous oestrogen, can bind to its receptors and develop the oestrogenic effect [9].

The therapeutic versatility of MP results from its complex phytochemical composition, which is characterised by several distinctive chemotypes. The plant contains a rich array of bioactive constituents, most notably alkylresorcinols, benzoquinone derivatives (particularly labisiaquinones), flavonoids, and triterpenoid saponins. Of particular significance are the alkylresorcinol derivatives and benzoquinone compounds, which appear to be characteristic features of MP and contribute significantly to its biological activities. For example, demethylbelamcandaquinone B [10] exhibits remarkable osteoanabolic properties, while labisiaquinone A and certain alkylresorcinols demonstrate potent cytotoxic activity against cancer cell lines [11].

Despite the increasing scientific literature on MP and its growing commercial application, there is still a significant gap in the reviews on this plant, particularly regarding its pharmacological properties and clinical potential. With the recent increase in MP-related research, a comprehensive review is essential to avoid future cross-studies and provide a clearer understanding of the medicinal properties of the plant. Therefore, the aim of this review is to identify and highlight the therapeutic benefits of MP from both experimental and clinical studies. The findings could help to provide a basis for the development of new complementary treatments for various diseases with MP.

## 2. Methods

A literature search was conducted to identify relevant articles related to the pharmacological potential of *Marantodes pumilum* (MP). Full text peer reviewed English articles were gathered from 2000 to December 2024 using electronic databases such as PubMed, Scopus, ScienceDirect, and Google Scholar. The search strategy used Boolean operators to combine the following terms: (“*Marantodes pumilum*” OR “*Labisia pumila*” OR “kacip fatimah”) AND (“pharmacology” OR “therapeutic” OR “medicinal” OR “phytochemical” OR “biological activity” OR “health benefits”). Additional searches were conducted by combining the plant terms with specific therapeutic areas such as osteoporosis, diabetes, cardiovascular health, wound healing, antimicrobial activity, and women’s health using the AND operator. The same Boolean logic was applied across all databases, with minor syntax adjustments made as required by individual database format. Our analysis of the published literature included data from in vitro, in vivo, and human studies.

## 3. The Botany of *Marantodes pumilum*

*Marantodes pumilum* (Blume) Kuntze (MP) is known as one of the flowering plants in the Primulaceae family. It is a small woody plant with creeping stems and long roots that bears lanceolate leaves and can grow up to 30 cm long and 13 cm wide. Some species have reddish-purple fruits and pink flowers [12]. MP is native to the tropical rainforests of Southeast Asia, particularly Malaysia, Thailand, Indonesia, the Philippines, and New Guinea. It thrives in specific ecological conditions: 80–100 m above sea level, 80% shade, 70% humidity, and temperatures of 25–35 °C.

**Figure 1:**
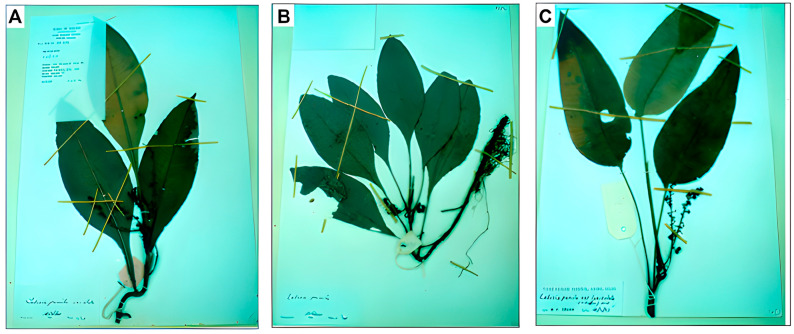
Voucher specimens of three main varieties of *Marantodes pumilum*: (**A**) *Marantodes pumilum* var. *alata*, (**B**) *Marantodes pumilum* var. *pumila,* and (**C**) *Marantodes pumilum* var. *lanceolata*. Specimens demonstrate the morphological differences used for variety identification in ethnobotanical and pharmacological studies. Picture adapted from Ibrahim et al. (2022) [3].

Of the eight recognised cultivars, three varieties, *MP* var. *pumila*, var. *alata*, and var. *lanceolata*, are widely distributed in Malaysian rainforests and represent the primary focus of scientific investigation [13]. These varieties exhibit distinct morphological characteristics: var. *pumila* has ovate leaf blades with margined petioles, var. *alata* has winged petioles with prominent red veins, while var. *lanceolata* possesses elongated, wingless petioles. However, the similar micromorphological features between the varieties pose significant taxonomic challenges, especially when the petioles are underdeveloped, making accurate field identification difficult [14]. Figure 1 shows voucher specimens of the three primary MP varieties [3].

The phytochemical composition of MP varies between different varieties and plant parts, which can significantly influence the therapeutic effects. While major phytochemical constituents remain generally consistent across varieties, their relative concentrations differ considerably, and these percentage variations can affect bioactivity and therapeutic efficacy. To address these sources of variability and improve reproducibility, the MP variety and plant part used are explicitly stated in this review when reported in the original studies. This approach allows researchers to identify the specific subspecies and plant constituent that exhibit particular therapeutic effects, thus providing readers with essential information for the accurate interpretation of pharmacological findings.

## 4. Traditional Use of *Marantodes pumilum*

The ethnomedicinal applications of MP reflect the traditional knowledge that has evolved over generations of observation and cultural transmission. Indigenous practitioners throughout the Malay Archipelago used this plant primarily for women’s reproductive health, including facilitating childbirth, postpartum recovery, uterine toning, and the management of menstrual disorders [15,16]. In addition to women’s health, MP has also played an important role in the traditional treatment of various conditions affecting multiple organ systems. Gastrointestinal applications included remedies for diarrhoea and flatulence. For inflammatory diseases, practitioners used MP preparations for rheumatism and fever. The plant also served as a traditional intervention for the treatment of infectious diseases, most notably gonorrhoea, and vascular diseases such as haemorrhoids [15,16]. Traditional healers prepared MP alone or in combination with complementary herbs as an alternative intervention for postmenopausal women at risk of osteoporosis due to oestrogen deficiency.

## 5. Phytochemistry of *Marantodes pumilum*

**Figure 2:**
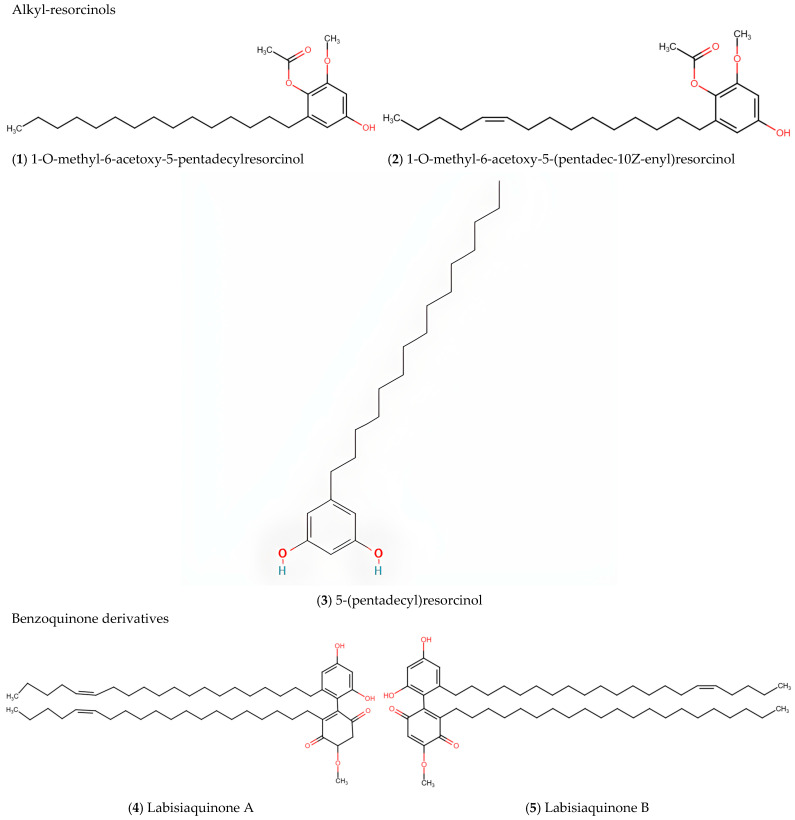

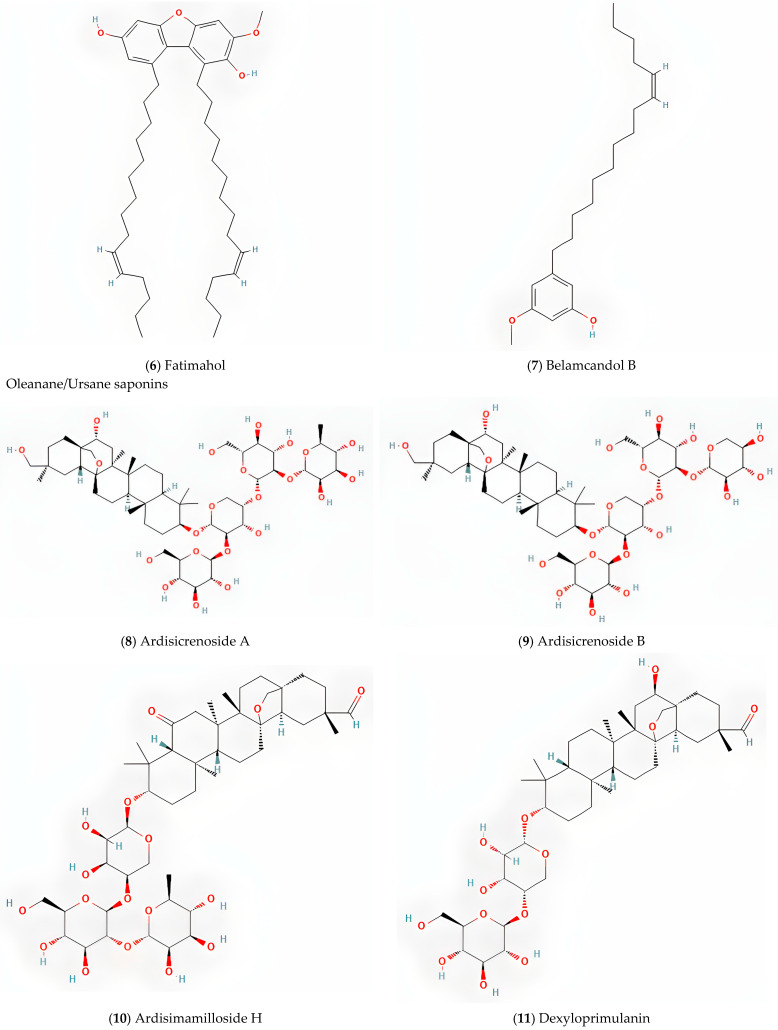
Chemical structures of major bioactive phytochemical compounds isolated and characterised from *Marantodes pumilum* based on the literature review, organised by chemical class. Alkyl-resorcinols: (**1**) 1-O-methyl-6-acetoxy-5-pentadecylresorcinol; (**2**) 1-O-methyl-6-acetoxy-5-(pentadec-10Z-enyl)resorcinol; (**3**) 5-(pentadecyl)resorcinol; Benzoquinone derivatives: (**4**) Labisiaquinone A; (**5**) Labisiaquinone B; (**6**) Fatimahol; (**7**) Belamcandol B; Oleanane/Ursane saponins: (**8**) Ardisicrenoside A; (**9**) Ardisicrenoside B; (**10**) Ardisimamilloside H; (**11**) Dexyloprimulanin.

MP contains contains several distinctive bioactive compounds that contribute to its diverse pharmacological activities. This section categorises these compounds by chemotype, with quantitative data provided where available. The phytochemical compounds are summarised in Table 1, while Figure 2 presents the chemical structures of major bioactive compounds isolated and characterised from MP.

### 5.1. Alkyl-Resorcinols

Alkyl-resorcinols constitute a significant chemotype characteristic of MP. Notable compounds in this class include 5-henicosene-1-yl-resorcinol [17], 1-O-methyl-6-acetoxy-5-(pentadec-10Z-enyl)resorcinol, 1-O-methyl-6-acetoxy-5-pentadecylresorcinol, 5-(pentadec-10Z-enyl)resorcinol, and 5-(pentadecyl)resorcinol [11]. The compounds 1-O-methyl-6-acetoxy-5-(pentadec-10Z-enyl)resorcinol and 1-O-methyl-6-acetoxy-5-pentadecylresorcinol demonstrate potent bioactivity with submicromolar growth inhibition (GI50) values against PC-3 (prostate cancer) and HCT-116 (colorectal cancer) cell lines [11]. The cytotoxic mechanism of alkyl-resorcinols in MP was elucidated by in vitro studies, which showed that 5-henicosene-1-yl-resorcinol induces apoptosis via the mitochondrial pathway [17].

### 5.2. Labisiaquinones and Benzoquinone Derivatives

MP contains unique benzoquinone derivatives including labisiaquinones A and B, which are characteristic compounds of this plant [11]. Demethylbelamcandaquinone B (Dmcq B), isolated from the leaf extract of MP var. *alata*, shows significant osteoanabolic activities comparable to 17β-oestradiol (E2) through the modulation of the BMP2 signalling pathway [10]. Other identified benzoquinone derivatives are fatimahol (di-alkenated dibenzofuran) [18], and belamcandol B [19], which contribute to the bioactivity profile of the plant.

### 5.3. Oleanane/Ursane-Type Saponins

Saponins are an important chemotype in MP, with the total content of saponins in the leaves being the highest in all varieties. With a 56.4 mg diosgenin equivalent/g dry weight, MP var. *pumila* has a significantly higher saponin content than var. *alata* (43.6 mg) and var. *lanceolata* (42.3 mg) [6]. The major triterpene saponins identified in MP include ardisicrenoside B, ardisiacrispin A, 3-O-α-L-rhamnopyranosyl-(1→2)-β-D-glucopyranosyl-(1→4)-α-L-arabinopynanosyl cyclamiretin A, and ardisimamilloside H [19]. Ardisiacrispin A, the major triterpenoid compound, shows considerable concentration variations of 0.11–2.46% in the different samples. The other saponins in MP include 13,28-epoxyoleanane glycoside [18].

### 5.4. Flavonoids

The flavonoids in MP are key contributors to its antioxidant activity and pharmacological effects. An HPLC analysis of the leaf extract of MP identified catechin, glycitin, rutin, naringin, and myricetin, while the stem extract contained genistin, naringin, and myricetin [20]. MP var. *pumila* generally exhibits a higher flavonoid content than var. *alata* and *lanceolata* [21]. A spectrophotometric analysis identified nine flavonols (quercetin, myricetin, kaempferol), two flavanols (catechin, epigallocatechin), and nine phenolic acids in the active 40% methanol fraction of the MP leaf extract [13]. CO_2_ enrichment studies show that an increased CO_2_ concentration from 400 to 1200 μmol mol^−1^ enhances the content of flavonoids including epicatechin, naringin, daidzein, quercetin, and myricetin in MP var. *alata* leaves [22].

### 5.5. Phenolic Compounds

Phenolic compounds are abundant in MP, with gallic acid being a predominant marker. The MP var. *pumila* leaf extract contains gallic acid at a concentration of 6.81 mg/g [23]. The total phenolic content in different varieties of MP ranges between 2.53 and 2.55 mg/g fresh weight [24]. The phenolic compounds identified in MP include pyrogallol, caffeic acid, and various phenolic acids, which contribute to the plant’s strong antioxidant activity [25].

### 5.6. Varietal Differences in Phytochemical Distribution

The phytochemical composition varies considerably between MP varieties and plant parts. MP var. *pumila* generally contains higher concentrations of bioactive compounds in both leaves and stems than var. *alata* and var. *lanceolata* [21]. Leaf extracts of all varieties have a higher bioactive content than stems and roots [6]. In leaf extracts, the phytochemical content follows the order var. *pumila* > var. *alata* > var. *lanceolata*, whereas in stem extracts, the pattern is var. *pumila* > var. *lanceolata* > var. *alata* [26]. The specific chemotype profile influences the biological activities of each variety, with var. *pumila* generally showing the strongest antioxidant and antimicrobial properties [6].

**Table 1:** Summary of MP Phytochemistry.

Chemotype	Representative Compounds	References
Alkyl-resorcinols	5-(pentadec-10Z-enyl)resorcinol, 5-(pentadecyl)resorcinol; 1-O-methyl-6-acetoxy-5-(pentadec-10Z-enyl)resorcinol; 1-O-methyl-6-acetoxy-5-pentadecylresorcinol; 5-(pentadec-10Z-enyl)resorcinol; 5-(pentadecyl)resorcinol	[11]
	5-Henicosene-1-yl-resorcinol	[17]
	Irisresorcinol (detected at concentrations as low as 0.2 μg/mL)	[19]
Benzoquinone derivatives	Demethylbelamcandaquinone B	[10,18]
	Labisiaquinone A and B	[11]
	Fatimahol (di-alkenated dibenzofuran)	[18]
	Belamcandol B	[19]
Oleanane/Ursane saponins	Dexyloprimulanin	[18]
	Ardisicrenoside B, Ardisiacrispin A, Ardisimamilloside H	[19]
Flavonoids	Naringin, Apigenin, Quercetin, Myricetin, Kaempferol	[6]
	(+)-Catechin; (−)-Epicatechin; Kaempferol-3-O-α-rhamnopyranosyl-7-O-β-glycopyranoside; Kaempferol-4′-O-β-glycopyranoside; Quercetin-3-O-α-rhamnopyranoside; Kaempferol-3-O-α-rhamnopyranoside	[11]
Phenolic acids	Gallic acid, Ellagic acid	[23]

## 6. Pharmacological Properties of *Marantodes pumilum*

In this section, the pharmacological properties of MP are discussed in relation to various characteristics. A total of fifty-two articles were found on the pharmacological properties of MP, including seventeen in vitro studies, thirty-five in vivo studies, and three human clinical trials.

### 6.1. Phytoestrogenic Effects of Marantodes pumilum

MP is widely recognised for its phytoestrogenic properties. Phytoestrogens are thought to require two hydroxyl groups and a phenolic ring to bind to oestrogen receptors [27]. Interestingly, the phytoestrogens in MP have these structural components, which allows them to bind to oestrogen receptors and potentially exert both oestrogenic and anti-oestrogenic effects [28]. In vitro studies have demonstrated that MP extracts contain compounds that exhibit selective binding profiles to oestrogen receptor subtypes. Through sequential extraction and receptor binding assays, researchers identified four alkylresorcinol derivatives and one dimeric 1,4-benzoquinone with specific binding affinities for both ERα and ERβ subtypes. These compounds demonstrated varying receptor selectivity, with IC50 values ranging from 4.7 to 453 μM for ERα and 5.1 to 86.7 μM for ERβ, suggesting a potential for tissue-selective oestrogenic activities [29].

**Figure 3:**
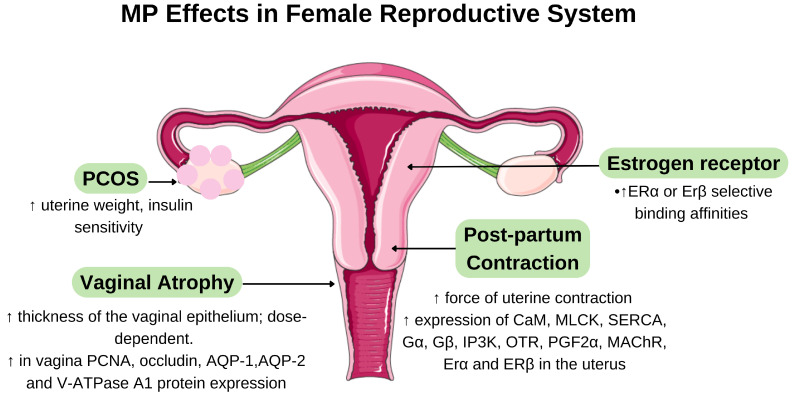
Proposed mechanism of *Marantodes pumilum* (MP)’s effects in the female reproductive system based on in vitro and in vivo studies. The diagram illustrates how MP extracts influence uterine contractility, ovarian function, and reproductive hormone pathways through modulation of various cellular targets and signalling cascades. ↑ indicates upregulation or increased activity. Abbreviations: AQP, aquaporin; PCNA, proliferating cell nuclear antigen; SERCA, sarco(endo)plasmic reticulum calcium-ATPases; Gα, G protein α; Gβ, G protein β; IP3K, inositol-1,4,5-trisphosphate 3-kinase; OTR, oxytocin-R antibody; PGF2A, prostaglandin (PGF)2α receptor; MAChR, muscarinic acetylcholine receptor.

The oestrogenic activity of MP was also confirmed using the Ishikawa cell line, an established model for the evaluation of oestrogenic compounds. The ethanol extract of MP roots significantly increased the activity of alkaline phosphatase (ALP), a marker for oestrogen receptor activation, and simultaneously inhibited cell proliferation [30]. This dual action suggests that MP compounds may act as selective oestrogen receptor modulators (SERMs) that exhibit tissue-specific agonist or antagonist properties. Because of these phytoestrogenic properties, MP compounds have been extensively investigated for the treatment of oestrogen deficiency conditions, particularly in postmenopausal symptoms, osteoporosis, cardiovascular disease, and oestrogen dysregulation disorders such as polycystic ovary syndrome (PCOS). These applications and their respective mechanisms of action are described in detail in the following sections. Figure 3 summarises the effects of MP on the female reproductive system.

### 6.2. Effects of MP on Postmenopausal Syndrome

Menopause is the result of declining ovarian function, which typically begins in the late 30s and peaks in the early 50s. This physiological transition leads to reduced oestrogen and progesterone production as the ovaries become less responsive to follicle stimulating hormone (FSH) and luteinising hormone (LH). The resulting hormonal imbalance manifests as menopausal symptoms including vasomotor symptoms (hot flushes, night sweats), sleep disorders, and joint pain. At the tissue level, diminished oestrogen increases vaginal pH, which leads to vaginal atrophy, characterised by thinning of the epithelium, reduced lubrication, and inflammation [31]. While conventional hormone replacement therapy (HRT) remains a primary treatment option, MP water extracts offer a promising alternative as they mimic the effect of oestradiol by binding to oestrogen receptors.

In a pivotal study using ovariectomized (OVX) rats, MP demonstrated hormonal effects comparable to oestrogen replacement therapy. The administration of MP increased oestradiol and testosterone levels while concurrently decreasing FSH and LH levels [32]. This hormonal modulation pattern mirrors the feedback mechanism observed with conventional HRT, indicating the ability of MP to restore hypothalamic–pituitary–gonadal axis function [32].

MP has also demonstrated efficacy in alleviating vaginal atrophy, a common postmenopausal condition. In ovariectomised rats, the intravaginal application of MP resulted in a significant increase in vaginal epithelial thickness compared to untreated controls. Mechanistically, MP enhanced vaginal epithelial cell proliferation through the upregulation of proliferating cell nuclear antigen (PCNA), improved lubrication by increasing aquaporin (AQP-1, AQP-2) expression, and restored vaginal acidity through the modulation of V-ATPase A1 protein levels [33]. These molecular changes correspond directly with the clinical improvements in vaginal tissue integrity and function, suggesting that MP may be an herbal alternative for the treatment of vaginal atrophy in postmenopausal women.

### 6.3. Effects on MP on Polycystic Ovary Syndrome and Postnatal Period

In addition to treating oestrogen deficiency, MP shows promise in the treatment of other reproductive disorders, particularly polycystic ovary syndrome (PCOS) and in supporting postpartum recovery. PCOS is a complex endocrine disorder characterised by elevated androgen levels, ovulatory dysfunction, and morphological abnormalities of the ovaries [34,35]. The pathophysiology of PCOS includes irregular ovulation, insulin resistance, obesity, and hyperinsulinemia, with elevated insulin levels amplifying ovarian androgen production [36]. The testosterone imbalance disrupts the gonadotropin-releasing hormone (GnRH) pathway, leading to increased LH and insufficient FSH secretion, which further exacerbates androgen excess and ovulatory failure [37].

In a DHT-induced rat model of PCOS, an MP extract improved insulin sensitivity and increased uterine weight, indicating a restoration of oestrogenic activity [38]. The improved insulin sensitivity appears to be due to the ability of MP to enhance glucose uptake by upregulating PPAR-γ in fat cells, which helps to reduce hyperinsulinaemia and subsequently decreases ovarian androgen production [39]. Lipid profiles were restored by lowering triglycerides and total cholesterol levels, consistent with the metabolic benefits of phytoestrogens. Complementing these findings, additional studies reported that MP significantly reduced inflammatory cytokines (TNF-α and C-reactive protein) associated with the development of osteoporosis in PCOS rats [40]. This anti-inflammatory effect occurs through the suppression of NF-κB signalling, which reduces inflammatory cytokines that normally worsen insulin resistance and promote androgen production. Both studies confirmed improved insulin sensitivity following MP treatment, highlighting its potential in managing PCOS-related metabolic disruptions through multiple pathways involving both hormonal modulation and anti-inflammatory action.

The administration of an aqueous MP extract significantly improved uterine contractility in the postpartum period through the coordinated upregulation of multiple contractile signalling pathways. Specifically, MP increased the expression of intracellular contractile proteins (calmodulin, myosin light-chain kinase, and sarco/endoplasmic reticulum Ca^2+^-ATPase) and uterotonin receptors (oxytocin receptor, prostaglandin F2α receptor, and muscarinic acetylcholine receptor) in the myometrium [41]. This enhancement of the contractile apparatus improves the ability of oestrogen to regulate contraction-associated proteins which facilitates uterine involution and recovery after childbirth. Table 2 summarises the phytoestrogenic properties of MP and its effect on postmenopausal syndrome, PCOS and postpartum.

### 6.4. Effects of MP on Bone Markers and Bone Density

Osteoporosis, defined by the World Health Organisation (WHO) as a decrease in bone mineral density (BMD) of more than 2.5 standard deviations, results from a pathological imbalance in bone remodelling. At the molecular level, the RANKL/RANK/OPG signalling axis serves as a central regulatory mechanism for bone homeostasis. The receptor activator of nuclear factor κB ligand (RANKL) binds to the RANK receptors on osteoclast precursors, promoting their differentiation, activation, and survival, while osteoprotegerin (OPG) acts as a decoy receptor that sequesters RANKL, thus inhibiting osteoclastogenesis [42].

In postmenopausal women, oestrogen deficiency disrupts this balance of bone remodelling by shifting the RANKL/OPG ratio towards increased RANKL expression, accelerating bone resorption and increasing the risk of osteoporosis. Oestrogen protects bones by binding to oestrogen receptors (ERs), particularly ERα, which is critical for bone health, while ERβ modulates the action of Erα [43]. While hormone replacement therapy (HRT) has been the conventional approach for managing this condition by directly modulating osteoclast activity, concerns about adverse effects have led to the exploration of alternatives. Soy isoflavones represent one such alternative, although their bone-protective effects are generally modest [44]. MP has emerged as another promising alternative, as it has been shown to upregulate oestrogen receptors, thereby attenuating oestrogen deficiency-induced bone loss and joint inflammation [10,45]. In addition to modulating receptors, MP counteracts three key pathological processes in postmenopausal osteoporosis: oxidative stress, inflammation, and dysregulated bone remodelling, which collectively contribute to accelerated bone resorption and impaired microarchitectural integrity [46,47].

Mechanistic studies have identified demethylbelamcandaquinone B (DmcqB), a compound isolated from MP, as a promoter of osteoblast differentiation. This compound activates the bone morphogenetic protein 2 (BMP2) pathway and upregulates the downstream transcription factor osterix (Osx) via oestrogen receptor signalling. The functional consequence of this molecular cascade is enhanced osteoblast activity, as evidenced by increased expression of bone formation markers including osteocalcin, elevated ALP activity, enhanced collagen synthesis, and accelerated calcium deposition. These in vitro findings have been confirmed in animal studies, where the administration of MP to ovariectomized rats significantly increased serum levels of osteocalcin (an osteoblast-specific protein) and simultaneously decreased the C-terminal telopeptide of type I collagen (CTX), a specific marker for bone resorption [48].

At the same therapeutic dose, MP modulates the RANKL/OPG balance by downregulating RANKL gene expression and simultaneously upregulating OPG and BMP-2 [49]. This dual mechanism of action effectively inhibits osteoclastogenesis by reducing RANKL-RANK interaction while promoting osteoblastogenesis by enhancing BMP-2 signalling. These findings establish that MP alleviates osteoporosis through the bidirectional modulation of bone remodelling, stimulating osteoblast-mediated bone formation while simultaneously suppressing osteoclast-mediated bone resorption. MP further amplifies its osteoanabolic effects through the activation of the canonical Wnt signalling pathway, a critical regulator of osteoblast differentiation and bone formation [50]. The administration of MP to oestrogen-deficient diabetic rats resulted in key components of this pathway, including Wnt3a, β-catenin, Frizzled (the Wnt receptor), Dishevelled (Dvl), and low-density lipoprotein receptor-related protein 5 (LRP-5) [50]. This coordinated upregulation of Wnt signalling components enhances the transcription of osteoblast-specific genes, thereby promoting osteoblastogenesis while inhibiting osteoclastogenesis.

Oestrogen acts as an antioxidant and its deficiency impairs the body’s defence against oxidative stress, which is one of the triggering factors for the increased bone resorption observed in postmenopausal osteoporosis. Studies have shown that the administration of MP successfully restored levels of antioxidant enzymes such as catalase (CAT), glutathione peroxidase (GPx) and superoxide dismutase (SOD) while significantly reducing malondialdehyde (MDA), a by-product of lipid peroxidation, in the femur of OVX rats [46]. These effects were found to be both dose- and time-dependent. Similar results were observed in oestrogen-deficient rats with diabetes mellitus, further emphasising the antioxidant potential of MP.

**Figure 4:**
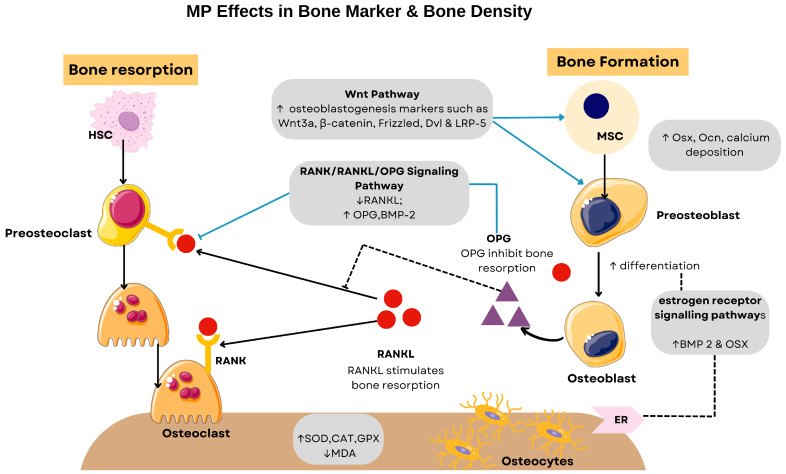
Proposed mechanisms of *Marantodes pumilum* (MP) effects on bone metabolism and osteoprotection. The pathway diagram summarises how MP extracts influence bone formation, resorption, and oxidative stress based on preclinical studies, showing interactions between osteoblast differentiation, osteoclast activity, and antioxidant pathways. Abbreviations: HSC, hematopoietic stem cell; RANK, receptor activator of nuclear factor kappa-B; WNT3A, wingless-related integration site 3A; DVL, dishevelled; LRP-5, low-density lipoprotein receptor-related protein 5; RANKL, receptor activator of nuclear factor kappa-B ligand; BMP-2, bone morphogenetic protein 2; OSX, osterix; OPG, osteoprotegerin; OCN, osteocalcin; SOD, superoxide dismutase; CAT, catalase; GPX, glutathione peroxidase; MDA, malondialdehyde; ER, oestrogen receptor. Arrows: ↑ increase, ↓ decrease.

In addition, oxidative stress and oestrogen deficiency trigger a pro-inflammatory state characterised by the increased production of osteoclastogenic cytokines including tumour necrosis factor-alpha (TNF-α), interleukin-1 (IL-1), and interleukin-6 (IL-6). MP attenuates this inflammatory cascade by restoring the balance of cytokine, potentially through direct oestrogen-like effects or through independent anti-inflammatory mechanisms [51]. In oestrogen-deficient rats with osteoarthritis induced by monosodium iodoacetate (MIA), MP also preserved joint integrity by modulating the expression of matrix metalloproteinase-13 (MMP-13) and its inhibitor, i.e., Tissue Inhibitor of Metalloproteinase-3 (TIMP-3) [45]. This modulation minimises cartilage degradation and reduces joint inflammation, which protects the articular structures from oestrogen deficiency-related damage [45]. Figure 4 illustrates the effects of MP in bone marker and bone density while Table 3 summarises the effects of MP on osteoporosis and osteoarthritis.

### 6.5. Effects of MP on Obesity and Cardiovascular System

Preclinical studies suggest that MP may influence metabolic regulation through interconnected pathways affecting glucose homeostasis and adipose tissue function. Animal studies have documented potential hypoglycaemic properties [59], acting synergistically with its effects on adipokine balance. For instance, MP administration to OVX rats showed promise in attenuating weight gain and restoring leptin/resistin ratios, hormones involved in satiety and insulin sensitivity regulation [58]. Further studies showed that MP downregulates the expression of hydroxysteroid (11-beta) dehydrogenase 1 (HSD11B1) and consequently lowers corticosterone levels [59]. This enzymatic modulation could potentially link MP’s metabolic effects with hormonal balance, as HSD11B1 functions as a cortisol activation regulator that influences visceral fat accumulation during oestrogen deficiency. By suppressing this enzymatic pathway, MP effectively creates a metabolic environment that is less conducive to the development of obesity despite low oestrogen levels.

Animal studies have also demonstrated potential cardiovascular benefits of MP through multiple mechanisms. Improvements in lipid profiles and reductions in hypercholesterolemia-induced oxidative stress observed in rat models suggest possible atheroprotective effects [5]. This vascular protection is physically manifested by a preserved architecture of the elastic lamellae and improved blood flow, structural changes that reduce the mechanical stress on the cardiovascular system. The antihypertensive effects observed in spontaneous hypertensive rats complement these vascular benefits, with the inhibition of calcium chloride-induced contraction by MP functioning via the soluble guanylate cyclase (sGC)/cyclic guanosine monophosphate (cGMP) pathway [60] to promote nitric oxide independent vasodilation. In acute cardiac injury models, MP administration was associated with reduced markers of cardiac damage (cTnI, CK-MB, LDH, ALT, AST) following induced myocardial infarction. While these preclinical findings suggest MP may offer cardiometabolic benefits through complementary pathways addressing both metabolic and cardiovascular aspects of oestrogen deficiency [61], translation to human therapeutic applications requires clinical validation. Table 4 summarises the effects of MP on cardiovascular system.

### 6.6. Effects of MP on Diabetes Mellitus

**Figure 5:**
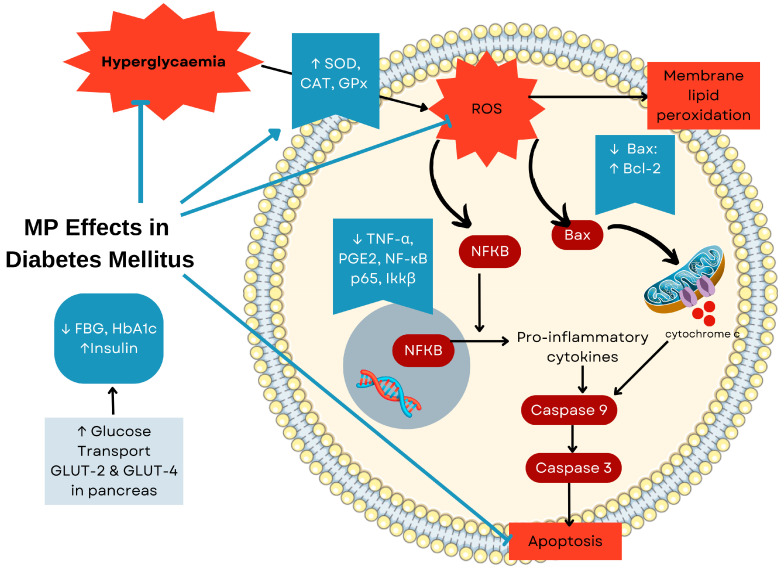
Proposed antidiabetic mechanisms of *Marantodes pumilum* (MP) based on in vitro and in vivo studies. The pathway diagram illustrates how MP extracts modulate glucose homeostasis through multiple mechanisms including enhanced glucose uptake, reduced inflammation, antioxidant activity, and prevention of β-cell apoptosis. The integrated pathways show MP’s potential in managing both insulin resistance and pancreatic dysfunction associated with diabetes mellitus. Abbreviations: HBA1c, glycated haemoglobin; GLUT, glucose transporter; SOD, superoxide dismutase; CAT, catalase; GPX, glutathione peroxidase; TNF-α, tumour necrosis factor alpha; PGE2, prostaglandin E2; NF-κB, nuclear factor kappa-B; IKKβ, IκB kinase beta; BAX, BCL-2-associated X protein; BCL, B-cell lymphoma; ROS, reactive oxygen species. Arrows: ↑ increase, ↓ decrease.

MP demonstrates antidiabetic effects through multiple interconnected mechanisms targeting both the primary pathology and secondary complications of diabetes. In streptozotocin-nicotinamide (STZ-NA)-induced diabetic rats, the aqueous extract of MP simultaneously reduced oxidative stress and inflammation by modulating the NF-κB signalling pathway. This anti-inflammatory action directly contributes to pancreatic protection, as evidenced by improved blood glucose regulation, enhanced insulin secretion, and preserved islet cell architecture [63]. The metabolic benefits of the extract go beyond pancreatic protection and improve peripheral glucose utilisation by upregulating the glucose transporters GLUT-2 and GLUT-4 in the liver and pancreatic islets [64,65,66], suggesting that its core anti-inflammatory and antioxidant properties provide systemic protection against diabetes-induced tissue damage. Figure 5 illustrates the integrated antidiabetic mechanisms of MP, while Table 5 summarises its effects on diabetes mellitus alongside other conditions.

### 6.7. Effects of MP on Wound Healing

MP varieties demonstrate significant wound-healing properties through multiple interconnected pathways (Table 6). These pathways involve the modulation of inflammatory responses, enhancement of cellular proliferation, extracellular matrix (ECM) remodelling, and antioxidant protection.

In a second-degree burn model, MP was shown to accelerate burn wound closure through enhanced collagen synthesis, as evidenced by the increased hydroxyproline content, a key amino acid in the collagen structure, increased fibroblast proliferation leading to improved ECM production, and improved neovascularization, which better oxygenated and nourished the healing tissue. This extract demonstrated efficacy comparable to silver sulfadiazine in second-degree burns [67]. Research has demonstrated that MP var. *pumila* and var. *alata* (1% and 2%) improved healing under low oestrogen conditions by increasing collagen deposition and fibroblast proliferation while reducing inflammatory cell infiltration in granulation tissue. Importantly, this study revealed that the wound-healing properties of MP act through oestrogen-independent mechanisms and not solely through phytoestrogen activity [68]. Further investigations by the same researchers on excised wounds confirmed these findings and clarified additional mechanisms [23]. Both MP variants accelerated re-epithelialisation while promoting the critical transition from collagen-III (early wound) to collagen-I (mature wound), indicating enhanced ECM remodelling. This process was supported by an increased fibronectin content, which serves as a scaffold for cellular migration and adhesion. In addition, the extracts upregulated antioxidant enzymes and decreased lipid peroxidation (LPO), suggesting the activation of the Nrf2 antioxidant pathway and likely due to the high phenolic content of MP [23].

Recent studies provided targeted mechanistic insights by isolating three bioactive compounds from MP var. *alata*: naringin, eicosan, and octacosan [69]. Naringin particularly enhanced fibroblast migration under both normal and insulin-resistant conditions and showed superior efficacy in diabetic wounds with improved epithelialisation and reduced inflammation. Molecular docking suggested that naringin interacts with matrix metalloproteinases (MMPs), regulating ECM remodelling during wound healing, which may explain its efficacy in diabetic wounds, which are typically characterised by dysregulated MMP activity [69].

The consistent efficacy of MP in different wound models (excision, burn, diabetes) and physiological conditions (normal, ovariectomised, diabetes) highlights its therapeutic potential, particularly for challenging conditions where conventional treatments are often of limited efficacy.

### 6.8. Effects of MP on Gout

The therapeutic potential of MP in gout management has been investigated in numerous studies targeting the dual pathophysiological mechanisms of the disease. Gout results from hyperuricaemia, which leads to the deposition of monosodium urate (MSU) crystals in the joints, triggering inflammatory cascades and painful arthritis. Studies have demonstrated that dichloromethane leaf extracts of MP exhibited significant xanthine oxidase (XO) inhibitory activity, with an IC50 value of 161.6 μg/mL [70]. Their phytochemical analysis led to the isolation of a novel compound, 3,7-dihydroxy-5-methoxy-4,8-dimethyl-isocoumarin, which showed remarkable XO inhibition, suggesting a potential application in the treatment of hyperuricaemia comparable to conventional XO inhibitors such as allopurinol [70]. The anti-hyperuricaemic efficacy of MP was further validated in vivo through investigations evaluating ethanolic extracts of leaves and roots of MP cultivars (var. *alata*, *pumila*, and *lanceolata*) in a potassium oxonate-induced hyperuricaemic rat model. Among these cultivars, the leaf extracts of var. *pumila* demonstrated superior XO inhibitory activity. The administration of these extracts to hyperuricaemic rats resulted in a time-dependent reduction in serum uric acid levels and suppressed hepatic XO activity, addressing the primary metabolic dysregulation in gout [4].

In addition to treating hyperuricaemia, MP has shown promising anti-inflammatory properties relevant to gout-associated arthritis. In a model in which MSU crystals were injected intra-articularly into the knee joints of rats, the extracts significantly mitigated the inflammatory response [4]. In vitro studies revealed that MP extracts inhibited the MSU crystal-induced secretion of key pro-inflammatory mediators including IL-1α, IL-1β, IL-8, TNF-α, and PGE2. This is particularly significant, as IL-1β is considered the main cytokine driving gout inflammation through the activation of the NLRP3 inflammasome. Corresponding in vivo findings confirmed reduced levels of IL-1α, IL-1β, IL-6, TNF-α, and PGE2 in the synovial fluid, suggesting efficacy in controlling the acute inflammatory phase of gout attacks.

Investigations exploring the immunomodulatory mechanisms of the plant reported that MP at a concentration of 50 μg/mL inhibited the lipopolysaccharide (LPS)-stimulated secretion of pro-inflammatory cytokines while paradoxically increasing PGE2 secretion [71]. This dual effect may offer therapeutic advantages, as certain PGE2 receptor subtypes may exert anti-inflammatory effects by suppressing neutrophil recruitment and promoting the resolution of inflammation, potentially facilitating recovery from acute gout flares. Collectively, these findings suggest that MP contains bioactive constituents that target both the metabolic and inflammatory components of gout pathophysiology, warranting further investigation for the potential development of novel therapeutics for gout. Table 6 summarises the effects of MP on gout.

**Table 6:** Effects of MP on wound healing and gout.

Extracts/Compound	Study Design	Treatment Dosage	Findings	References
Wound Healing				
MP var. *alata* leaf and root aqueous extraction	In vivo: 50 male Sprague-Dawley rats with second-degree burn wounds (80 °C steel rod, 10 s)	2% MP ointment for 3 weeks	(1) ↑ Hydroxyproline content → ↑ collagen synthesis → improved structural integrity; (2) ↑ fibroblast proliferation → ↑ ECM production → accelerated granulation tissue formation; (3) ↑ neovascularization → ↑ oxygen/nutrient delivery → ↑tissue regeneration→ significant reduction in burn wound size	[67]
MP var. *alata* and var. *pumila* leaf and root extract aqueous extraction	In vivo: 126 male Sprague-Dawley rats with excisional wounds (6 mm diameter, 2 mm thickness)	1% and 2% of MP ext for 13 days	(1) ↑ Collagen-III to collagen-I transformation → enhanced tensile strength; (2) ↑ fibronectin → improved scaffold for cellular migration and attachment; (3) high phenolics→ ↑ antioxidant enzyme activities + ↓ lipid peroxidation → reduced oxidative damage→ accelerated re-epithelialisation and improved wound closure rate	[23]
MP var. *alata* dicholoromethane extraction Isolated compounds: naringin (NAR), eicosan (EIC), and octacosan (OCT)	In vitro HDF in normal and insulin-resistant conditions In vivo 36 diabetic SD rats with excisional wounds (3 wounds with 5 mm thickness)	10 g of each compound for 30 days	(1) High radical scavenging against DPPH, NO, OH•, and O_2_• → reduced oxidative stress; (2) NAR → ↑fibroblast migration in both normal and insulin-resistant conditions; (3) NAR → regulated ECM degradation/remodelling; (4) NAR> EIC/OCT for epithelialisation, fibroblast proliferation, ↓ inflammation	[69]
MP var. *pumila* and *alata root* and leaf extraction	In vivo: 99 ovariectomized Sprague-Dawley rats with full-thickness excisional wounds (6 mm diameter)	1% and 2% of MP ext for 13 days	(1) ↑ collagen content and fibroblast cells → enhanced structural regeneration; (2) ↓ inflammatory cells in granulation tissues → ↓scarring and scar width →↑ healing rate	[68]
Gout				
MP leaves and roots of dichloromethane and methanol extraction	In vitro xanthin oxidase (XO) assay	400 μg/mL for extracts and 100 μg/mL for isolated compound	3,7-dihydroxy-5-methoxy-4,8-dimethyl-isocoumarin → extremely potent XO inhibition (IC50 = 0.66 ± 0.01 μg/mL) → potential specific biochemical target	[4,70]
MP leaves and roots ethanol extraction	In vitro Xanthine Oxidase Inhibition Assay In vivo 36 SD rats with induced hyperuricemia and MSU crystal-induced inflammation	200 mg/kg/day orally for 14 days	(1)↓ serum uric acid levels and hepatic XO activity; (2) Inhibition of MSU crystal-induced inflammatory cytokines (IL-1α, IL-1β, IL-8, TNF-α, PGE2) → ↓ inflammatory mediators in synovial fluid	[4]
MP leaves and roots dichloromethane methanol and water extraction	In vitro cytokine and PGE2 assay	50 µg/mL	Inhibited LPS-induced pro-inflammatory cytokines (IL-1α, IL-1β, IL-6, IL-8, TNF-α) while ↑ PGE2 secretion → differential regulation of inflammatory response pathways	[71]

Abbreviations: SD: Sprague-Dawley, ECM: extracellular matrix, HDF: human dermal fibroblasts, DM: diabetes mellitus, STZ: streptozotocin, DPPH: 2,2-diphenyl-1-picrylhydrazyl, NO: nitric oxide, OH•: hydroxyl radical, O_2_•: superoxide radical, MMP: matrix metalloproteinase, XO: xanthine oxidase, MSU: monosodium urate, IL: interleukin, TNF-α: tumour necrosis factor alpha, PGE2: prostaglandin E2, LPS: lipopolysaccharide. Arrows: ↑ indicates increase; ↓ indicates decrease; → indicates the sequential flow or progression.

### 6.9. Effects of MP on Cancer

MP has an anti-cancer potential that has been demonstrated in multiple cell lines and animal models and acts mainly through three interrelated pathways: the induction of apoptosis, regulation of cell cycle, and modulation of redox balance (Table 7). A study on SK-UT-1 uterine leiomyosarcoma cells showed that MP ethanol/water extracts at certain concentrations reduced the number of viable cells by up to 61.64%, primarily through the induction of apoptosis [72]. This finding was further reinforced in an in vivo uterine fibroid xenograft model, where treatment with MP led to a significant reduction in tumour volume over three weeks [72]. The cytotoxic effects of MP also extend to other cancers, including human melanoma cells (HM3KO), where the ethanol extract exhibited potent cytotoxicity (IC50 of 16.18 μg/mL) through the activation of the intrinsic (mitochondrial) apoptotic pathway [7]. This activation occurs via a cascade that begins with the upregulation of p53. Increased p53 then increases the expression of the pro-apoptotic protein Bax while simultaneously decreasing the anti-apoptotic protein Bcl-2, resulting in an increased Bax/Bcl-2 ratio. This shift in the ratio destabilises the integrity of the mitochondrial membrane and triggers the release of cytochrome c and subsequent caspase activation, which ultimately leads to programmed cell death. At the same time, MP extracts cause cell cycle arrest in the G1 phase and thus prevent DNA replication and cell division [7]. The cytotoxic activity of MP extends to multiple cancer types, including breast (MCF-7), colon (HCT-116), and prostate (PC-3) cell lines [11]. Phytochemical analysis has identified specific bioactive compounds responsible for this activity, in particular labisiaquinone A and 5-(pentadec-10Z-enyl) resorcinol. These compounds belong to the class of quinones and alkylresorcinols, which generally exert anti-cancer effects via different mechanisms. Quinones can participate in the redox cycle and generate reactive oxygen species that selectively damage cancer cells, whose antioxidant defences are often weakened. With their amphipathic structure, alkylresorcinols can disrupt the integrity of cell membranes and impair the function of mitochondrial. The observed cytotoxicity even at low concentrations (0.1–100 μM) suggests high specificity for the targets of cancer cells [11]. Table 7 summarises the anti-cancer effects of MP.

### 6.10. Antimicrobial Properties of MP

The rapid adaptation of pathogens to antibiotics and antifungals has caused concern, especially in view of the emergence of multidrug-resistant organisms. This has prompted the search for alternative therapeutic approaches, including the identification of potential antibacterial properties of natural compounds. Medicinal plants have attracted attention due to their antimicrobial properties and offer safe, natural, and cost-effective alternatives to synthetic antimicrobials in the face of increasing antibiotic resistance [73]. Despite these challenges, medicinal plants show great potential in combating antibiotic resistance, as various studies have reported significant antibacterial activities. Further research is needed to confirm the efficacy, safety, and mechanisms of these bioactive compounds [74].

Several bioactive molecules with antimicrobial properties have been identified in MP flavonoids such as kaempferol, apigenin, naringin, and quercetin, which not only exhibit antioxidant properties, but also have antimicrobial activity against bacteria such as *Staphylococcus aureus*, *Staphylococcus epidermis*, *Bacillus cereus*, *Bacillus subtilis*, *Pseudomonas aeruginosa*, *Klebsiella pneumoniae*, *Salmonella typhi,* and *Escherichia coli* [73]. Phenolic compounds such as gallic acid, saponins, and triterpenoids also show antimicrobial effects against both Gramme-positive and Gramme-negative bacteria [75]. In this section, the antimicrobial properties of MP are discussed, with a focus on antibacterial, antifungal, and antiviral activities.

#### 6.10.1. Antibacterial Activity

The antibacterial properties of MP were first documented through the testing of leaf extracts against both Gramme-positive and Gramme-negative bacteria [76]. Although most extracts demonstrated antibacterial activity, the methanol extract was ineffective against *Staphylococcus aureus*. Subsequent comparative studies evaluated the antibacterial activity of methanol extracts from different plant parts (leaves, stems, roots) across three MP varieties (*pumila*, *alata*, *lanceolata*) [6]. The *pumila* variety exhibited moderate to significant activity, particularly against Gramme-positive bacteria, with leaf extracts showing the highest efficacy. HPLC analysis revealed elevated flavonoid concentrations in the *pumila* variety, which contributed to its superior antibacterial properties [6].

Phytochemical investigations have identified twenty-one known metabolites and three novel compounds in MP root extracts [18]. Notably, this represents the only study in which individual MP compounds were evaluated for antimicrobial activity, emphasising the antimicrobial potential of belamcandol B and 1,3-dihydroxy-5-[10(Z)-pentadecenyl]benzene (synonym to 5-((Z)-pentadec-10-enyl)resorcinol). There has been no study investigating the mechanism of action of the compounds against known pathogens. Based on the mechanistic configuration, 1,3-dihydroxy-5-[10(Z)-pentadecenyl]benzene is classified as an alkyl resorcinol derivative. Resorcinol derivatives are known to penetrate microbial membranes, destabilise lipid bilayers, and increase the permeability of the bacterial cell membrane [77]. Belamcandol B, on the other hand, contains an alkyl chain and phenolic hydroxyl group. The alkyl chain could disrupt the cell wall of the pathogen due to the hydrophobicity of the chain [78,79]. The phenolic hydroxyl group mediates antimicrobial activity by damaging the bacterial cell membrane through the formation of hydrogen bonds with its membrane proteins and phospholipids, inducing oxidative stress in the microbe, and inhibiting the activity of intracellular enzymes [80].

Conflicting results have been reported regarding antibacterial activity, with some studies finding no observable effects, possibly due to lower extract concentrations used [81]. However, these investigations provided valuable insights into the mechanism of action against *Escherichia coli*, suggesting that the MP extract reduces intracellular bacterial load through apoptotic effects on bladder epithelial cells. The apoptotic effects cause bladder epithelial cells to disrupt the protected intracellular environment that bacteria utilise to evade neutrophil phagocytic activity and form intracellular bacterial communities (IBCs). Comparative studies of three MP varieties demonstrated that the pumila variety exhibited the strongest antibacterial effect, particularly against Gramme-positive bacteria. This enhanced efficacy was attributed to the high content of phenols and flavonoids characteristic of this variety [82]. Phenols (such as salicylic acid, syringic acid, gallic acid, and pyrogallol) and flavonoids (such as apigenin, quercetin, kaempferol, naringin, and myricetin) are known for their antimicrobial effect by altering and damaging the bacterial cell wall and cell membrane, inhibition of nucleic acid synthesis, reduction in biofilm formation, disruption of the multidrug efflux system, and induction of apoptosis [6,80,83]. Saponins (such as ardisiacrispin and asdisimamilloside) have been reported to damage the integrity of bacterial cell membranes, inhibit biofilm formation, induce stress responses and cell autolysis, and inhibit efflux pumps [84,85].

#### 6.10.2. Antifungal and Antiviral

Although the antifungal properties of MP are less extensively researched, there are indications that its extracts may be effective against fungal pathogens. The activity of leaf extracts against two fungal species has been demonstrated [6]. However, contradictory findings have been reported, with some studies finding no antifungal activity while others reported weak to moderate antifungal effects, emphasising the variability in results [81]. The observed antifungal properties of MP are hypothesised to be related to the presence of phenolic acids and flavonoids, which are recognised for their natural fungicidal activity [6,26]. The antifungal activity of phenolic acids and flavonoids has been attributed to their ability to disrupt the cell membrane, activate the mitochondrial antioxidant system, inhibit nucleic acid synthesis, induce apoptosis, inhibit fungal metabolism, and downregulate virulence factors [80,86,87]. Although empirical studies on the antiviral properties of MP are lacking, recent computational modelling has suggested potential antiviral applications. An in silico investigation demonstrated that apigenin, a compound present in MP, exhibits potential antiviral activity against Marburg virus infection [88]. This theoretical finding indicates that further research is warranted to investigate the antiviral potential of MP compounds through experimental validation.

Antiviral properties of apigenin have already been reported against enterovirus 71, herpes simplex virus (HSV) -1 and -2, hepatitis C virus, influenza virus, hand-foot-and-mouth disease virus, and African swine fever virus by inhibiting viral replication, reactive oxygen species formation, and cellular apoptosis [89]. Future research should investigate the synergistic potential of MP compounds in combination with standard antimicrobial agents, nanoparticles, or other natural products to increase antimicrobial efficacy, reverse resistance, and reduce cytotoxicity. The antimicrobial potential of MP makes it a promising candidate for the development of new antimicrobial agents. Table 8 summarises the antimicrobial effects of MP.

### 6.11. Clinical Studies

Clinical studies investigating MP have yielded promising health benefits, although the evidence base remains limited (Table 9). A critical evaluation of the available human trials reveals both promising results and important methodological considerations.

A 16-week randomised, double-blind, placebo-controlled study evaluated 197 women (pre and postmenopausal) taking 400 mg/day of MP. This study demonstrated significant improvements in cognitive function (memory, concentration), menopausal symptoms (vasomotor symptoms, sleep quality), and anxiety, particularly in premenopausal women. While no significant changes were observed in FSH, LH, or oestradiol levels, a positive trend was observed for oestradiol. Cardiovascular benefits included significant reductions in total cholesterol and LDL, particularly in women with high triglycerides [90]. However, limitations included the relatively short intervention period and the lack of standardisation of dietary and lifestyle factors that could influence the outcomes. Safety monitoring confirmed no significant adverse events, and liver function tests and hormone profiles remained within normal ranges throughout the intervention period.

A longer-term six-month study of 63 postmenopausal women receiving 280 mg/day of MP reported a significant reduction in triglycerides (1.4 vs. 1.9 mmol/L) and minor improvements in fasting glucose and total cholesterol [8]. The strength of this study was the longer duration of the intervention and the focus on a specific demographic, although the modest sample size limited the statistical power for secondary outcomes. In addition, although the reduction in triglycerides was statistically significant, it remained within the normal physiological range.

More recently, a four-month study of 142 participants with obesity investigated the effects of 375–750 mg of MP extract (SKF7) daily (NCT05851599) [90]. The results showed a significant reduction in body weight, waist circumference (WC), and weight-to-height ratio (WHtR), with improvements associated with the gradual increase in the SKF7^®^ dose. The safety analysis revealed minimal adverse effects: <1.5% of participants had liver enzyme and complete blood count abnormalities, and adverse effects were observed in less than 38% of participants. Key limitations included the relatively short duration of the intervention and the undisclosed exact composition of the standardised extract, which limited the assessment of long-term efficacy.

Current knowledge gaps include the need for larger, longer-term studies (>1 year) to determine safety profiles, optimal dosing regimens, and the durability of effects. The standardisation of MP extracts in the various studies is inconsistent, making the direct comparison of efficacy difficult. In addition, mechanistic studies linking bioactive components to specific physiological metabolic pathways are needed to better understand the mode of action of MP.

Future clinical research on MP should prioritise addressing these gaps through well-designed studies that include (1) larger sample sizes with sufficient statistical power; (2) standardised extract preparations with consistent bioactive compounds; (3) safety monitoring including liver function tests and hormone profiles; (4) longer intervention periods (≥12 months) to determine the durability of effects; and (5) investigation of potential interactions with conventional medications.

Overall, while current evidence suggests the therapeutic potential of MP, particularly in metabolic and menopausal symptoms, further well-designed clinical trials with adequate safety monitoring are required before definitive clinical recommendations can be made.

## 7. Conclusions

In summary, studies suggest that MP has potential therapeutic applications in managing osteoporosis, osteoarthritis, diabetes mellitus, and cardiovascular conditions, while also showing promise in wound healing, gout management, and cancer prevention. However, it is crucial to emphasise that most of these findings derive from animal and in vitro studies, with limited clinical validation in humans.

Several research gaps warrant consideration for future investigations. The mechanisms underlying the therapeutic effects of MP need further investigation, particularly with regard to interactions with hormone receptors and long-term effects on chronic disease. Given the proven anti-obesity and antidiabetic properties of MP, an unexplored area of great interest is its potential effects on the central nervous system, which is not discussed in this review. The link between metabolic dysfunction and neurological function suggests that MP may affect neural pathways involved in appetite regulation, glucose homeostasis, and cognitive function. The hypothalamic–pituitary axis, which regulates both metabolic and reproductive functions, is a particularly relevant target for the phytoestrogenic compounds of MP. Additionally, the current research is characterised by methodological heterogeneity, with significant variability in MP preparation methods, extraction techniques, and dosing protocols across studies. While this diversity reflects the exploratory nature of natural product research, it poses a challenge for establishing optimal therapeutic protocols and allowing for direct comparisons between studies.

This comprehensive review, drawing from multiple databases and diverse study types, provides a foundation for advancing MP research from preclinical potential to clinical application. The substantial body of evidence documented supports the rationale for prioritising well-designed clinical trials with standardised MP preparations, appropriate control groups, and sufficient follow-up periods. Future investigations should focus on elucidating molecular signalling pathways, optimising dosing regimens, evaluating long-term safety profiles, and exploring potential interactions with conventional medications. Additionally, computational studies including molecular docking analyses would provide valuable insights into the binding interactions between MP bioactive compounds and their target receptors, particularly oestrogen receptors, to better understand the mechanistic basis of observed therapeutic effects. Particular attention should be given to investigating the potential neuroprotective and neuromodulatory effects of MP, including its influence on neurotransmitter systems, neuroinflammation, and cognitive function, especially in the context of metabolic syndrome and age-related neurodegeneration. As interest in MP continues to grow within integrative medicine approaches, the diverse pharmacological activities presented in this review highlight MP’s significance as a promising therapeutic agent deserving of continued scientific investigation.

## Figures and Tables

**Table 2:** Phytoestrogenic properties of MP and its effect on postmenopausal syndrome, PCOS, and postpartum.

Extracts/Compound	Study Design	Dosage	Findings	Refs.
Phytoestrogenic Properties				
MP var. *alata* aqueous -methanolic extract	In vitro PolarScreen™ ER Alpha and Beta Competitor Assay Kit	100, 30, 10, 3, 1, 0.3, 0.1 μg/mL	4 alkylresorcinol derivatives and belamcandol B → varying affinities for ERα (IC50: 4.7–453 μM) and ERβ (IC50: 5.1–86.7 μM) → demonstrating distinct phytoestrogenic activity with receptor subtype preferences	[29]
MP var. *pumilum* root and leaf aqueous extraction	In vitro Ishikawa cells (human endometrial adenocarcinoma cells)	100 µg/ mL	Tissue-selective oestrogen action → ↑ ALP activity with simultaneous ↓ proliferation of cells → suggesting mixed agonist/antagonist effects on uterine tissue	[30]
Postmenopausal Syndrome				
MP whole plant aqueous extraction	In vivo 30 ovariectomised Sprague-Dawley rats	17.5, 35.0, and 70.0 mg/kg/day orally for 60-day	Restoration of oestrogen-dependent parameters → ↑ oestrogen and testosterone levels with ↓ FSH and LH levels	[32]
MP whole plant aqueous extraction	In vivo 36 ovariectomised Sprague-Dawley rats	10, 25, and 50 mg intravaginal gel preparation; treated for 7 days	Dose-dependent ↑ in vaginal epithelium thickness → ↑ expression of proliferation marker (PCNA), tight junction protein (occludin), and water/ion transporters (AQP-1, AQP-2, V-ATPase A1) → alleviation of vaginal atrophy symptoms	[33]
PCOS				
MP var. *alata* aqueous extraction	In vivo 22 female Wistar rats; 7 weeks of DHT exposure, implanted subcutaneously in the neck with a 90-day continuous-release pellet	50 mg/kg/day: orally for 3.5 weeks	↑ uterine weight (restored oestrogenic activity) and insulin sensitivity → altered adipokine balance (↑ plasma resistin levels, ↓ mRNA expression of leptin in adipose tissue) → improved lipid profile→ metabolic and reproductive improvements	[38]
MP aqueous extraction	In vivo 36 Sprague-Dawley rats; PCOS group fed with HFD for 90 days	25 and 50 mg/kg/day; stomach gavage for 90 days	Anti-inflammatory and hormonal regulation → ↑ oestradiol levels and insulin sensitivity → ↓ inflammatory markers (C-reactive protein and TNF-α) → improved PCOS parameters	[40]
MP var. *alata* aqueous extraction	In vitro 3T3-L1 adipocyte cell line In vivo 22 female Wistar rats	50 mg/kg/day: orally for 4 weeks	PPARγ expression (40% mRNA upregulation) and protein levels → ↑ insulin sensitivity (35% improvement) and glucose uptake in adipocytes → improved glucose homeostasis via euglycemic-hyperinsulinemic clamp → metabolic improvements in PCOS	[39]
Postpartum				
MP var. *alata* aqueous extraction	In vivo 24 day-1 post-delivery Sprague-Dawley female rats	100, 250, and 500 mg/kg/day; orally for 7 days	Dose-dependent ↑ in uterine contraction force → ↑ key contractility proteins and receptors (CaM, MLCK, SERCA, Gα, Gβ, IP3K, OTR, PGF2α, MAChR, ERα and ERβ) despite ↓ oestradiol levels → improved postpartum uterine tone	[41]

ALP: alkaline phosphatase (enzyme measured to assess oestrogen activity); FSH: follicle-stimulating hormone; LH: luteinizing hormone; DHT: dihydrotestosterone; PCNA: proliferating cell nuclear antigen (proliferation marker); AQP-1, AQP-2: aquaporin water channel proteins; V-ATPase A1:vacuolar-type proton pump; TNF-α: tumour necrosis factor alpha (inflammatory marker); HFD: high-fat diet; CaM: calmodulin; MLCK: myosin light-chain kinase; SERCA: sarcoplasmic reticulum calcium ATPase; Gα, Gβ: G protein subunits; IP3K: inositol triphosphate kinase; OTR: oxytocin receptor; PGF2α: prostaglandin F2α receptor; MAChR: muscarinic acetylcholine receptor; ↑ indicates increase; ↓ indicates decrease; → indicates the sequential flow or progression.

**Table 3:** Effects of MP on osteoporosis, osteoarthritis, diabetes mellitus, and cardioprotective effects.

Extracts/Compound	Study Design	Treatment Dosage	Findings	References
Osteoporosis				
MP var. *alata* crude aqueous extract and fractionated with hexane, dichloromethane and methanol solvents.	In vitro cell culture: Murine pre-osteoblastic MC3T3-E1 cells	10–100 µg/mL	DmcqB activates ERα/ERβ → ↑ osteoblastic gene expression (BMP2, Osx, Ocn) → ↑ collagen synthesis, ALP activity and calcium deposition while modulating RANKL/OPG ratio (↓RANKL, ↑OPG) → inhibit osteoclastogenesis	[10]
MP var. *alata* root aqueous extraction	In vivo 32 ovariectomised Wistar rats	17.5 mg/kg/day orally for 8 weeks	Stimulates osteoblast activity (↑ObS/BS, OS/BS, OV/BV) and inhibiting osteoclast function (↓OcS/BS, ES/BS) → improves dynamic bone formation parameters and bone microarchitecture	[52]
MP var. *alata* whole plant aqueous, ethanol and methanol extraction	In vivo 48 ovariectomised Sprague-Dawley rats	20 or 100 mg/kg/day orally for 9 weeks.	Phytoestrogens enhance trabecular microstructure → denser trabecular network with ↑ connectivity density, bone volume (BV/TV), and trabecular number (TbN) with ↓ trabecular separation (TbSp) and structure model index (SMI) → improved bone quality	[47]
MP var. *alata* leaves and roots aqueous extraction	In vivo 30 ovariectomised Sprague-Dawley rats	20 mg/kg/day orally for 8 weeks	Plant flavonoids and saponins enhance mineral deposition → ↑ trabecular densities, bone volume, cortical thickness (Ct.Th), connectivity density (Conn.D), and bone mineral density (BMD) → improved mechanical properties (↑ maximum force and stress)	[53]
MP var. *alata* root aqueous extraction	In vivo 32 ovariectomised Wistar rats	17.5 mg/kg/day orally for 8 weeks	Bioactive compounds shift bone turnover balance → ↑ osteocalcin (bone formation marker) with ↓ CTX (bone resorption marker) → net bone formation over resorption	[48]
MP var. *alata* root aqueous extraction	In vivo 32 ovariectomised Wistar rats	17.5 mg/kg/day orally for 8 weeks	Phytoestrogens → enhanced biomechanical competence with improved stress values, strain values, and Young’s modulus through modulation of collagen cross-linking	[54]
MP var. *alata* whole plant aqueous extraction	In vivo 96 ovariectomised Sprague-Dawley rats	20 or 100 mg/kg/day orally for 3, 6, or 9 weeks.	Time-dependent enhancement of bone matrix quality → gradual ↑ in maximum load, stress value, stiffness value, and Young’s modulus (elasticity) → reflecting development of mature bone	[55]
MP var. *alata* root aqueous extraction	In vivo 32 ovariectomised Wistar rats	17.5 mg/kg/day orally for 2 months	Modulation of RANKL/OPG/BMP-2 signalling axis → prevention of RANKL gene elevation and maintaining OPG and BMP-2 gene expression → transcriptional control of bone remodelling	[49]
MP var. *alata* whole plant aqueous extraction	In vivo 96 ovariectomised Sprague-Dawley rats	20 or 100 mg/kg/day orally for 3, 6, or 9 weeks.	Phenolic compounds → ↑ SOD and GPx with ↓ LPO (MDA) in femur→ ↓ oxidative stress-induced osteoblast apoptosis	[45]
MP var. *alata* Whole plant aqueous extraction	In vivo 96 ovariectomised Sprague-Dawley rats	20 or 100 mg/kg/day orally for 3, 6, or 9 weeks.	Dose and time-dependent modulation of bone remodelling → improved trabecular network with ↑ bone volume and trabecular number (TbN) and ↓ trabecular separation (TbSp) → sustained restoration of bone mass	[56]
MP var. *alata* Leaves aqueous extraction	In vivo 42 ovariectomised Sprague-Dawley rats and induction of DM using combination of STZ at 55 mg/kg (i.p.) and nicotinamide at 100 mg/kg	50 or 100 mg/kg/day orally for 28 days	Dual targeting of glycaemic control and bone metabolism via (1) NF-κB pathway inhibition → ↓ inflammatory markers (NF-κB p65, IKKβ, IL-6, IL-1β) (2) Nrf2 activation → ↑ antioxidant enzymes (NQO-1, HO-1, SOD, CAT) (3) RANKL/OPG modulation and enhancement of osteogenic factors (BMP-2, Type-1 collagen, Runx2) → improved bone quality	[51]
MP var. *alata* Leaves aqueous extraction	In vivo 42 ovariectomised Sprague-Dawley rats and induction of DM using combination of STZ at 55 mg/kg (i.p.) and nicotinamide at 100 mg/kg	50 or 100 mg/kg/day orally for 28 days	Activation of canonical Wnt/β-catenin signalling → ↑ pathway components (Wnt3a, Frizzled, Dvl, LRP5) → enhanced osteoblast proliferation (↑ PCNA, c-Myc) and ↓ apoptosis (↓ caspase-3/9, Bax) → improved bone regeneration and collagen content	[50]
Osteoarthritis				
MP leaves Ethanol-aqueous extraction	In vivo 40 ovariectomised Sprague-Dawley rats and induction of OA by injecting mono-iodoacetate into the right knee joints	150 or 300 mg/kg/day, orally for 8 weeks	Suppression of cartilage degradation pathways → ↓ catabolic enzymes (MMP-13) and chondrocyte hypertrophy markers (RUNX2, COL10a1) and ↓ apoptosis (↓ CASP3) → preserved cartilage integrity and improved chondrocyte morphology	[45]
MP leaves Ethanol-aqueous extraction	In vivo 40 ovariectomised Sprague-Dawley rats and induction of OA by injecting mono-iodoacetate into the right knee joints	150 or 300 mg/kg/day, orally for 8 weeks	Multi-target chondroprotection through: (1) ↓ inflammatory mediators (NO, PTGS2) and ↑ IL-10 → anti-inflammatory action (2) ↓ collagenases (MMP-1/3) and proteoglycan release→ matrix preservation (3) ↓ trabecular spacing, porosity, and cartilage fissures → relatively intact cartilage surfaces→ structural maintenance	[57]
MP var. *alata* aqueous extraction	In vivo 42 ovariectomised Sprague-Dawley rats	10, 20, or 50 mg/kg/day, orally for 30 days	Adipokine modulation → ↑ leptin and ↓ resistin levels → improved metabolic profile with ↓ weight gain and preserved uterus weight ratio→ mitigating obesity-related bone loss	[58]

ALP: alkaline phosphatase; Bax: Bcl-2-associated X protein; BMD: bone mineral density; BMP-2: bone morphogenetic protein 2; BV/TV: bone volume/tissue volume; CAT: catalase; CASP3: caspase-3; COL10a1: collagen type X alpha 1; Conn.D: connectivity density; Ct.Th: cortical thickness; CTX: C-terminal telopeptide; DM: diabetes mellitus; Dvl: dishevelled protein; ERα/ERβ: oestrogen receptor alpha/beta; ES/BS: eroded surface/bone surface; GPx: glutathione peroxidase; HO-1: heme oxygenase 1; IKKβ: inhibitor of nuclear factor kappa-B kinase subunit beta; IL-1β: interleukin-1 beta; IL-6: interleukin-6; IL-10: interleukin-10; LPO: lipid peroxidation; LRP5: low-density lipoprotein receptor-related protein 5; MDA: malondialdehyde; MMP-1/3: matrix metalloproteinase 1/3; MMP-13: matrix metalloproteinase 13; NF-κB: nuclear factor kappa-B; NO: nitric oxide; NQO-1: NAD(P)H quinone dehydrogenase 1; Nrf2: nuclear factor erythroid 2-related factor 2; OA: osteoarthritis; ObS/BS: osteoblast surface/bone surface; Ocn: osteocalcin OcS/BS: osteoclast surface/bone surface; OPG: osteoprotegerin; OS/BS: osteoid surface/bone surface; Osx: osterix; OV/BV: osteoid volume/bone volume; PCNA: proliferating cell nuclear antigen; PTGS2: prostaglandin-endoperoxide synthase 2 (COX-2); RANKL: receptor activator of nuclear factor kappa-B ligand; Runx2: runt-related transcription factor 2; SMI: structure model index; SOD: superoxide dismutase; STZ: streptozotocin; TbN: trabecular number; TbSp: trabecular separation. Arrows: ↑ indicates increase; ↓ indicates decrease; → indicates the sequential flow or progression.

**Table 4:** Effects of MP on cardiovascular system.

Extracts/Compound	Study Design	Treatment Dosage	Findings	References
Cardioprotective Effects				
MP var. *alata* aqueous extraction	In vivo 42 ovariectomised Sprague-Dawley rats	10, 20, or 50 mg/kg/day, orally for 30 days	↓ HSD11B1 mRNA and protein in liver and adipose tissues → ↓ cortisol activation → ↓visceral adiposity and metabolic risk	[59]
MP var. *alata* aqueous extraction	In vivo 35 ovariectomised Sprague-Dawley rats	17.5 mg/kg/day, orally for 3 months	maintained the elastic lamellae architecture with significant aortic wall thickness→ enhanced vascular integrity and compliance through balanced elastin-collagen metabolism	[62]
MP var. *alata* hydroalcoholic extraction	In vivo 54 male Wistar rats and induction of myocardial infarction using isoproterenol (10 mg/mL; s.c.) to the rats on day 30 and 31	100, 200, or 400 mg/kg/day orally for 28 days	(1) ↑ GPx, CAT, SOD → ↓ free radical damage→ ↓ oxidative stress; (2) membrane stabilisation → improved cell integrity → ↓ cardiac injury markers (cTnI, CK-MB, LDH, ALT, AST) → preserved cardiomyocyte function	[61]
MP var. *alata* Hydroalcoholic extraction	In vivo 54 male Wistar rats fed with 2% cholesterol diet for 8 weeks	100, 200, or 400 mg/kg/day orally for 4 weeks	(1) Improved lipid metabolism → ↓ TC, TG, LDL; ↑ HDL and atherogenic indices; (2) enhanced antioxidant defence → ↓ oxidative stress and MDA; (3) ↓ AST, ALT, LDH and atheroma lesions in abdominal aorta → vascular and hepatic protection	[5]
MP aerial and leaf Multiple solvent extraction	In vivo 36 male spontaneously hypertensive rats (SHRs)	500 mg/kg/day orally for 28 days	Calcium-dependent vasodilation: (1) calcium channel antagonism → ↓ KCl-induced contraction; (2) inhibition of Ca^2+^ release from sarcoplasmic reticulum → ↓ intracellular Ca^2+^; (3) endothelium-independent vasorelaxation → ↓ SBP through direct action on vascular smooth muscle	[60]

ALT: alanine aminotransferase AST: aspartate aminotransferase CAT: catalase CK-MB: creatine kinase-MB cTnI: cardiac troponin I GPx: glutathione peroxidase HDL: high-density lipoprotein HSD11B1: 11β-hydroxysteroid dehydrogenase type 1 LDH: lactate dehydrogenase LDL: low-density lipoprotein MDA: malondialdehyde MP: *Marantodes pumilum* SBP: systolic blood pressure SHRs: spontaneously hypertensive rats SOD: superoxide dismutase TC: total cholesterol TG:tTriglycerides var.: variety s.c.: subcutaneous. Arrows: ↑ indicates increase; ↓ indicates decrease; → indicates the sequential flow or progression.

**Table 5:** Effects of MP on diabetes mellitus.

Extracts/Compound	Study Design	Treatment Dosage	Findings	References
Diabetes Mellitus				
MP 50% aqueous ethanol extraction	In vivo 30 STZ-induced (60 mg/kg) diabetic SD rats with diabetic neuropathy	150 or 300 mg/kg/day orally for 10 weeks	(1) ↓ NF-κB pathway activation → ↓ TNF-α, PGE2 → reduced neuroinflammation; (2) ↓ oxidative stress → preserved neuronal integrity → improved behavioural outcomes→ these mechanisms collectively led to neuroprotection with histologically confirmed preservation of peripheral nerves	[65]
MP 50% aqueous ethanol extraction	In vivo 30 STZ-induced (60 mg/kg) diabetic SD rats with diabetic retinopathy	150 or 300 mg/kg/day orally for 10 weeks	(1) ↓ Inflammatory cascade → ↓ TNF-α, PGE2 → reduced retinal inflammation; (2) regulation of vascular permeability → ↓ claudin-1, VEGF → preserved blood-retinal barrier; (3) antioxidant effects → ↓ MDA/glutathione ratio → ↓oxidative damage→ maintained retinal structure with reduced vascular leakage	[66]
MP var. *alata* leaves aqueous extraction	In vitro Glucose uptake in 3T3-L1 cell line In vivo 42 male SD rats and DM induction using 55 mg/kg STZ and 110 mg/kg NA	250 or 500 mg/kg/day for 28 days	(1) Enhanced insulin signalling pathway → ↑ glucose uptake in adipocytes → improved glycaemic control (↓ FBG, HbA1c); (2) ↑ insulin production, ↑ HDL, ↑ antioxidant enzymes; (3) ↓ NF-κB p65, ↓ Ikkβ, ↓ TNF-α → reduced tissue inflammation; (4) ↑ Bcl-2/↓ Bax ratio, ↓ caspase-9 → preserved β-cell mass and function→ pancreas protection effect	[64]
MP var. *alata* aqueous leaf and ethanol (50%) stem-root extracts	In vivo 54 ovariectomised SD rats and DM induction using of 30 mg/kg kg and 110 mg/kg NA	50 or 100 mg/kg/day for 28 days	Insulin sensitisation pathway → ↑ p-IRS1 → ↑ PI3K → ↑ p-Akt → ↑ GLUT-2/4 expression → enhanced glucose utilisation and improved tolerance; (2) inhibition of inflammatory signalling → ↓ p-IKKβ → ↓ pNF-Kβ → ↓ iNOS, COX2 → reduced inflammatory damage; (3) protection of β-cells → ↓ caspase 3 and cleaved caspase 3 → preserved islet architecture and function	[63]

Abbreviations: STZ: streptozotocin, NA: nicotinamide, FBG: fasting blood glucose, TNF-α: tumour necrosis factor alpha, PGE2: prostaglandin E2, MDA: malondialdehyde, VEGF: vascular endothelial growth factor, NF-κB: nuclear factor kappa-B, IRS: insulin receptor substrate, PI3K: phosphoinositide 3-kinase, Akt: protein kinase B, GLUT: glucose transporter, iNOS: inducible nitric oxide synthase, COX2: cyclooxygenase-2, IKK: IκB kinase, Bcl-2: B-cell lymphoma 2, Bax: Bcl-2-associated X protein. Arrows: ↑ indicates increase; ↓ indicates decrease; → indicates the sequential flow or progression.

**Table 7:** Anti-cancer effects of MP.

Anti-Cancer				
Extracts/Compound	Study Design	Treatment Dosage	Findings	References
MP whole plant ethanol/water extraction	In vitro Uterine leiomyosarcoma SK-UT-1 cells In vivo 24 uterine fibroid xenograft in athymic mouse model	In vitro 100 and 250 μg/mL In vivo 200 or 400 mg/kg/day for 3 weeks	In vitro induce apoptosis → ↓ viable cell up to 61.64% In vivo ↓ average tumour volume	[64]
MP whole plant hexane, ethanol, and water extraction	In vitro Human melanoma HM3KO cells	0–5 mg/mL	(1) ↑ p53, Bax, Bax/Bcl-2 ratio; ↓ Bcl-2 → mitochondrial membrane destabilisation → cytochrome c release → caspase activation →apoptosis; (2) arrest at G1 phase → prevented DNA replication and proliferation	[7]
MP leaves Methanol/water extraction	In vitro Breast (MCF-7), colon (HCT-116), and prostate (PC-3) cells	0.1 and 100.0 uM	1-O-methyl-6-acetoxy-5-(pentadec-10Z-enyl)resorcinol (1) and 1-O-methyl-6-acetoxy-5-pentadecylresorcinol (4) → ↑ submicromolar growth inhibition with selective cytotoxicity against PC-3 and HCT-116→ likely disrupt cell membrane integrity and interfere with mitochondrial function	[11]
MP var. *pumila* methanol extraction	In vitro PC3 and LNCaP prostate cancer cell line		5-henicosene-1-yl-resorcinol (alkyl-resorcinol) → (1) mitochondrial pathway: ↑Bax, ↓Bcl-2 → mitochondrial membrane depolarisation → ↑caspases 3/7 activation → nuclear DNA fragmentation; (2) anti-migration pathway: ↓ALOX-5 → ↓VEGF-A, ↓CXCL12 → inhibited cell migration/invasion→ induced apoptosis	[17]

p53: tumour protein p53; Bax: Bcl-2-associated X protein; Bcl-2: B-cell lymphoma 2; ALOX-5: arachidonate 5-lipoxygenase; VEGF-A: vascular endothelial growth factor A; CXCL12: C-X-C motif chemokine 12. Arrows: ↑ indicates increase; ↓ indicates decrease; → indicates the sequential flow or progression.

**Table 8:** Antimicrobial effects of MP.

Extract/Compound	Study Design	Treatment Dosage	Organisms Tested	Findings	References
MP leaves acetone, methanol, ethanol, and phosphate buffer pH 7 extraction	In vitro Agar-well diffusion method	5–100 mg/mL	*Staphylococcus aureus*	MIC ranging from 25 to 75 mg/mL	[6]
			*Pseudomonas aeruginosa*	MIC of 25 mg/mL	
MP var. *pumila*, *alata*, *lanceolata* leaves, stems, and roots methanol extraction	In vitro Disc diffusion method	0–500 µg/well	*Micrococcus luteus*	Zone of inhibition ranging from 0.33 to 0.65 cm; effective but less potent than the standard agent	[6]
			*Bacillus subtilis* B145	Zone of inhibition ranging from 0.79 to 1.15 cm; effective but less potent than the standard agent	
			*Bacillus cereus* B43	Zone of inhibition ranging from 0.73 to 1.1 cm; effective but less potent than the standard agent	
			*Staphylococcus aureus* S1431	Zone of inhibition ranging from 0.29 to 0.95 cm; effective but less potent than the standard agent	
			*Enterobacter aerogenes*	Zone of inhibition ranging from 0.71 to 1.23 cm; effective but less potent than the standard agent	
			*Klebsiella pneumonia* K36	Zone of inhibition ranging from 0.62 to 1.12 cm; effective but less potent than the standard agent	
			*Escherichia coli* E256	Zone of inhibition ranging from 0.45 to 1.32 cm; effective but less potent than the standard agent	
			*Pseudomonas aeruginosa* PI96	Zone of inhibition ranging from 0.33 to 0.75 cm; effective but less potent than the standard agent	
	In vitro Agar well diffusion method	0–500 µg/well	*Fusarium* sp.	Zone of inhibition ranging from 0.30 to 0.75 cm; weak to moderate antifungal activities compared to the standard agent	
			*Candida* sp.	Zone of inhibition ranging from 0.40 to 0.79 cm; weak to moderate antifungal activities compared to the standard agent	
			*Mucor* sp.	Zone of inhibition ranging from 0.28 to 0.69 cm; weak to moderate antifungal activities compared to the standard agent	
MP root methanol extraction	In vitro Broth dilution method	0–100 μg/mL	*Staphylococcus aureus*	IC_50_ of 3.13 μg/mL; mild antibacterial activity of 1,3-dihydroxy-5-[10(Z)-pentadecenyl]benzene compound	[18]
			Methicillin resistant *S. aureus*	IC_50_ ranging from 0.83 to 19.41 μg/mL; effective antibacterial activity of belamcandol B and 1,3-dihydroxy-5-[10(Z)-pentadecenyl]benzene compound but less potent the standard agent	
			*Escherichia coli*,	No inhibitory activity	
			*Pseudomonas aeruginosa*	No inhibitory activity	
			*Mycobacterium intracellulare*	No inhibitory activity	
			*Candida albicans*	No inhibitory activity	
			*Candida glabrata*	No inhibitory activity	
			*Candida krusei*	No inhibitory activity	
			*Cryptococcus neoformans*	No inhibitory activity	
			*Aspergillus fumigatus*	No inhibitory activity	
MP aqueous extraction	In vitro Agar-well diffusion method	1000 µg/mL	Uropathogenic *Escherichia coli* (UPEC) strain CFT073	No inhibitory activity; reduced bacterial load in the bladder epithelial cell (BEC) infection model via BEC apoptosis	[81]
			*Proteus mirabilis*	No inhibitory activity	
			*Pseudomonas aeruginosa*	No inhibitory activity	
			*Staphylococcus saprophyticus*	No inhibitory activity	
			*Candida albicans*	No inhibitory activity	
MP var. *pumila*, *alata*, *lanceolata* leaves, and roots aqueous extraction	In vitro Agar-well diffusion method	450 µg/well	*Fusarium* sp.	Zone of inhibition ranging from 0.45 cm to 0.71 cm; weak to moderate antifungal activities compared to the standard agent	[26]
			*Candida* sp.	Zone of inhibition ranging from 0.51 cm to 0.82 cm; weak to moderate antifungal activities compared to the standard agent	
			*Mucor* sp.	Zone of inhibition ranging from 0.33 cm to 0.81 cm; weak to moderate antifungal activities compared to the standard agent	
MP var. *pumila*, *alata*, *lanceolata* leaves aqueous extract	In vitro Disc diffusion method	300 µg/disc	*Staphylococcus aureus* S1431	Zone of inhibition ranging from 0.55 to 0.70 cm; effective but less potent than the standard agent	[82]
			*Bacillus subtilis* B145	Zone of inhibition ranging from 0.65 to 0.75 cm; effective but less potent than the standard agent	
			*Bacillus cereus* B43	Zone of inhibition ranging from 0.60 to 0.75 cm; effective but less potent than the standard agent	
			*Enterococcus aerogenes*	Zone of inhibition ranging from 0.40 to 0.63 cm; effective but less potent than the standard agent	
			*Escherichia coli* E256	Zone of inhibition ranging from 0.78 to 0.90 cm; effective but less potent than the standard agent	
			*Pseudomonas aeruginosa* PI96	Zone of inhibition ranging from 0.30 to 0.40 cm; effective but less potent than the standard agent	
			*Micrococcus luteus*	Zone of inhibition ranging from 0.40 to 0.55 cm; effective but less potent than the standard agent	
			*Klebsiella pneumonia* K36	Zone of inhibition ranging from 0.55 to 0.65 cm; effective but less potent than the standard agent	

**Table 9:** Clinical studies on MP.

Extracts/Compound	Study Design	Treatment Dosage	Findings	References
MP water extract	A pilot study: randomised, double-blind, placebo-controlled conducted amongst postmenopausal Malay women (N = 63)	280 mg/day MP extract for 6 months	Lower fasting glucose, total cholesterol, LDL, HDL, triglycerides, LH, and oestradiol levels; no effect on hormonal profile ↓ adjusted mean triglyceride level (1.4 mmol/L) vs. placebo group (1.9 mmol/L)	[8]
MP var. *alata* water extract	A randomised, double-blind, placebo-controlled, parallel group, 16-week study in healthy pre- and postmenopausal women aged 40–60 years old (N = 197)	400 mg/day for 16 weeks	Women’s Health Questionnaire: Significant improvements in memory, concentration, vasomotor symptoms, and sleep. No significant changes in FSH, LH, or oestradiol levels ↓ in total cholesterol and LDL-C, especially among participants with elevated triglycerides	[90]
SKF7^®^ (a patented standardised extract of MP)	A randomised, double-blind, multicentric, placebo-controlled, phase 2 dose-ranging evaluation of SKF7^®^ in obese (N = 133) (NCT05851599)	375, 562.5, and 750 mg day for 16 months	Dose dependent reduction in abdominal obesity; ↓ in BW, BMI, WC, and WHtR	[91]

BMI: body mass index BW: body weight FSH: follicle-stimulating hormone HDL: high-density lipoprotein LDL: low-density lipoprotein LDL-C: low-density lipoprotein cholesterol LH: luteinizing hormone MP: *Marantodes pumilum* n/N: number of participants var.: variety WC: waist circumference WHtR: waist-to-height ratio. Arrows: ↑ indicates increase; ↓ indicates decrease.

## Data Availability

All the data are contained within the article.
